# Synergistic Effects of Microencapsulated Polyphenols and Concurrent Training on Metabolic Health and Fitness in Overweight/Obese Adults with Prediabetes

**DOI:** 10.3390/nu17213358

**Published:** 2025-10-25

**Authors:** Udomlak Sukatta, Prapassorn Rugthaworn, Ketsaree Klinsukhon, Piyaporn Tumnark, Nattawut Songcharern, Yothin Teethaisong, Yupaporn Kanpetta, Jatuporn Phoemsapthawee

**Affiliations:** 1 Kasetsart Agricultural and Agro-Industrial Product Improvement Institute, Kasetsart University, Bangkok 10900, Thailand; aapuls@ku.ac.th (U.S.); aappsr@ku.ac.th (P.R.); aapkrk@ku.ac.th (K.K.); 2 Department of Sports Science, Faculty of Sports and Health Science, Kasetsart University, Nakhon Pathom 73140, Thailand; piyaporn.t@ku.th; 3 Exercise Science and Sports Performance, Faculty of Physical Therapy and Sports Medicine, Rangsit University, Pathum Thani 12000, Thailand; nattawut.s@rsu.ac.th; 4 Department of Medical Sciences, Faculty of Allied Health Sciences, Burapha University, Chon Buri 20131, Thailand; yothin.te@go.buu.ac.th; 5 Physical Education Program, Faculty of Liberal Arts and Management Science, Kasetsart University Chalermphrakiat Sakon Nakhon Campus, Sakon Nakhon 47000, Thailand; yupaporn.ka@ku.ac.th

**Keywords:** anthocyanin, hyperglycemia, insulin, polyphenols, inflammation, oxygen consumption

## Abstract

**Background/Objectives**: Prediabetes markedly increases the risk of progression to type 2 diabetes. While exercise and dietary polyphenols independently enhance metabolic health, their combined and synergistic effects remain unclear. This randomized, single-blind, placebo-controlled trial investigated the synergistic effects of concurrent training and a microencapsulated persimmon–karonda polyphenol formulation on glycemic control and inflammatory outcomes in adults with prediabetes and who are overweight/obese. **Methods**: Forty-three participants completed the intervention and were assigned to placebo, concurrent training (CBT), supplementation (EATME), or the combined intervention (CBT + EATME) for 8 weeks. Primary outcomes included fasting blood glucose (FBG), glycated hemoglobin (HbA1c), homeostatic model assessment for insulin resistance (HOMA-IR), high-sensitivity C-reactive protein (hs-CRP), interleukin-6 (IL-6), tumor necrosis factor-alpha (TNF-α), adiponectin, physical fitness, and quality of life (QoL). **Results**: All intervention groups (CBT, EATME, and CBT + EATME) showed improvements in glycemic indices, with the greatest reductions in FBG (*p* < 0.01), HbA1c (*p* < 0.05), and HOMA-IR (*p* < 0.01) observed in the CBT + EATME group compared with placebo. All interventions significantly reduced hs-CRP (*p* < 0.01) and IL-6 (*p* < 0.01), accompanied by marked increases in adiponectin (*p* < 0.01), compared with placebo. In the CBT + EATME group, reductions in hs-CRP were positively correlated with improvements in HOMA-IR (*r* = 0.627, *p* < 0.05). Both CBT and CBT + EATME improved muscular strength and maximal oxygen consumption (*V̇*O_2_max), with the combined intervention producing greater gains in upper- and lower-body strength (*p* < 0.05), *V̇*O_2_max (*p* < 0.05), and the psychological well-being domain of QoL (*p* < 0.05) compared with placebo. **Conclusions**: These findings highlight that combining concurrent training with microencapsulated polyphenol supplementation produced the most consistent improvements across metabolic, inflammatory, and fitness outcomes, supporting this combined approach as an integrated and synergistic strategy to reduce diabetes risk and promote overall health in at-risk adults. The trial was registered at the Thai Clinical Trials Registry (TCTR20250512003).

## 1. Introduction

Prediabetes and the increasing number of overweight adults are major global health concerns that substantially contribute to the rising prevalence of type 2 diabetes mellitus (T2DM) [1]. Both conditions are characterized by metabolic dysregulation and chronic low-grade inflammation, which represent key mechanisms driving the development of insulin resistance [1,2]. In individuals with excess adiposity, hypertrophic adipose tissue releases pro-inflammatory cytokines—such as tumor necrosis factor-alpha (TNF-α), interleukin-6 (IL-6), and C-reactive protein (CRP)—that impair insulin signaling and exacerbate both metabolic and vascular dysfunction [1,3,4]. In parallel, levels of adiponectin—an adipocyte-derived hormone with insulin-sensitizing, anti-inflammatory, and anti-atherogenic properties—are typically reduced in obesity and prediabetes [5,6]. Together, this pro-inflammatory and hypoadiponectinemic milieu accelerates the progression from prediabetes to overt T2DM and heightens the risk of atherosclerosis and other cardiovascular complications.

Bioactive compounds from natural sources, particularly polyphenols, have gained considerable attention for their ability to modulate metabolic and inflammatory pathways [7,8]. Persimmon (*Diospyros kaki* L.) and karonda (*Carissa carandas* L.), two Asian fruits traditionally valued for nutritional and medicinal purposes, are rich in diverse polyphenols such as quercetin, ferulic acid, and chlorogenic acid, and are especially abundant in cyanidin-3-glucoside, a potent anthocyanin with strong antioxidant and anti-inflammatory activity [9,10]. These polyphenols have been associated with improved glycemic control and lower inflammation, consistent with evidence that they enhance metabolic regulation and antioxidant capacity [11,12,13]. Moreover, cyanidin-3-glucoside, the predominant anthocyanin, has been shown to enhance vascular function by supporting endothelial health [14,15].

Despite their promising bioactivity, the application of polyphenols in functional foods is limited by their instability when exposed to light, temperature fluctuations, oxygen, and pH changes, leading to diminished activity during processing and storage. Furthermore, their inherently low bioavailability restricts efficient absorption and utilization in vivo [16]. Microencapsulation offers a promising strategy to overcome these limitations by protecting active compounds from degradation, enabling controlled release, and improving bioavailability [17]. Microencapsulation using maltodextrin has been shown to improve the stability, solubility, and gastrointestinal absorption of polyphenols by protecting them from oxidation and degradation during digestion [18,19]. The maltodextrin-based system used in this study was selected for its high encapsulation efficiency and demonstrated ability to enhance polyphenol bioaccessibility. This technology also allows targeted delivery, thereby maximizing biological efficacy and providing opportunities to integrate polyphenol supplementation with lifestyle interventions, such as exercise training, to achieve synergistic health benefits [16,17].

Exercise is a cornerstone strategy for improving metabolic health and physical function in individuals with prediabetes and who are overweight/obese; however, the magnitude of adaptation can be modulated by nutritional factors. Increasing scientific interest has focused on whether polyphenol supplementation can augment the metabolic and fitness adaptations induced by exercise training through mechanisms involving enhanced antioxidant defense, attenuation of inflammation, and improved vascular function [20,21,22]. Concurrent training, which integrates aerobic and resistance exercise, has demonstrated superior effects on glycemic control, body composition, and inflammatory status compared with either modality alone [23,24]. Meta-analyses further confirm that concurrent training reduces insulin resistance—as assessed by the homeostasis model assessment of insulin resistance (HOMA-IR)—and lowers glycated hemoglobin (HbA1c) and C-reactive protein (CRP) in individuals with T2DM and who are overweight/obese [25,26]. Notably, exercise and polyphenolic compounds share overlapping targets related to energy metabolism and cellular stress responses, providing a rationale for their potential synergistic effects [27]. Nevertheless, few studies have investigated the combined effects of concurrent training and polyphenol-rich supplementation in individuals with prediabetes and who are overweight/obese.

Beyond its clinical relevance, validation of persimmon and karonda as functional food ingredients may contribute to sustainable health-oriented innovation by linking local agricultural resources with nutritional strategies for metabolic improvement. Accordingly, the present study evaluated the efficacy of a microencapsulated persimmon–karonda formulation combined with concurrent training on glycemic control, inflammatory markers, and cardiometabolic risk factors in adults with prediabetes and who are overweight/obese. This integrative approach demonstrates how dietary supplementation and exercise can be synergistically applied to support disease prevention and health promotion. To our knowledge, this is among the first pilot trials to examine the combined effects of microencapsulated polyphenol supplementation and concurrent training in individuals with prediabetes, addressing an important gap in evidence regarding integrated lifestyle–nutraceutical strategies in this at-risk population.

## 2. Materials and Methods

### 2.1. Study Design

This study was an 8-week, randomized, single-blind, controlled clinical trial conducted at the Faculty of Sports and Health Science, Kasetsart University, Thailand, between June and October 2024. A four-arm, parallel-group design was used to evaluate the independent and combined effects of concurrent training and a microencapsulated polyphenol-rich formulation containing persimmon and karonda extracts on glycemic control, inflammatory markers, and cardiometabolic outcomes in adults with prediabetes and overweight. The protocol complied with the principles of the Declaration of Helsinki and was approved by the Institutional Review Board of the Kasetsart University Research Ethics Committee (approval No. COA67/045; 7 May 2024). The trial was prospectively registered in the Thai Clinical Trials Registry (TCTR20250512003).

**Figure 1:**
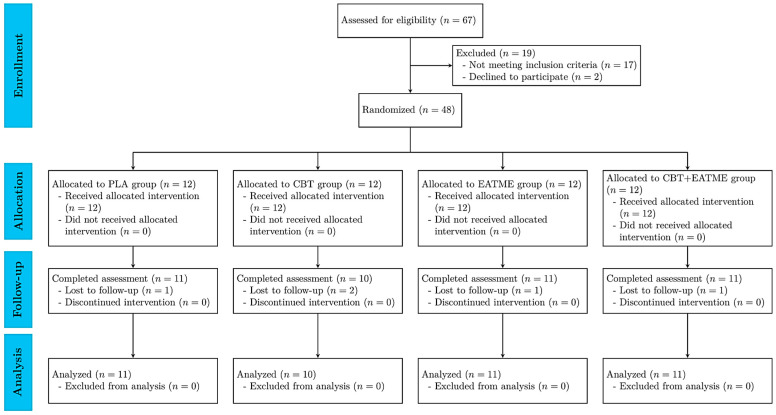
Consort flow diagram of the study.

Study recruitment and reporting followed the Consolidated Standards of Reporting Trials (CONSORT) guidelines [28], as shown in Figure 1, with the completed checklist provided in Supplementary Table S1. A total of 67 individuals were assessed for eligibility, of whom 48 met the inclusion criteria and were randomized into four groups: (i) placebo control (PLA; *n* = 12); (ii) concurrent training (CBT; *n* = 12); (iii) microencapsulated polyphenol supplementation (EATME; *n* = 12); and (iv) combined concurrent training with supplementation (CBT + EATME; *n* = 12). Reasons for exclusion included not meeting prediabetes criteria (*n* = 17) and declining participation (*n* = 2). During the 8-week intervention, five participants withdrew due to personal scheduling conflicts. No adverse events, allergic reactions, or exercise-related injuries were reported in any group throughout the study.

The intervention lasted 8 weeks, with participants completing two assessments: a baseline evaluation within one week prior to initiation and a post-intervention evaluation conducted at least 72 h after the final exercise session. Intervention and safety analyses were performed according to a modified intention-to-treat principle. Participant involvement was limited to providing feedback on communication and adherence monitoring during the intervention. All data were collected at the Faculty of Sports and Health Science, Kasetsart University. To minimize diurnal variation, all testing was conducted at the same time of day (±1 h) under standardized laboratory conditions (room temperature 25 ± 2 °C; humidity 48 ± 5%).

### 2.2. Participants

#### 2.2.1. Sample Size Calculation

An a priori power analysis was performed using G*Power version 3.1 (Heinrich Heine University, Düsseldorf, Germany) to determine the required sample size. Because no prior studies have examined the combined effects of polyphenol supplementation and CBT in individuals with prediabetes, the effect size estimate was derived from a nutraceutical trial investigating nano-curcumin supplementation in metabolic syndrome (MetS), which reported a large metabolic effect (f = 0.77) [29]. We acknowledge that this represents an upper-bound estimate and may overstate the expected effects in the present four-arm, 8-week design. Assuming α = 0.05, statistical power = 0.80, and two repeated measurements (baseline and post-intervention), the required sample size was 32 participants. To accommodate an anticipated dropout rate of 20%, the target sample size was increased to 40 participants (approximately 10 per group).

#### 2.2.2. Inclusion and Exclusion Criteria

Eligible participants were adults aged 25–59 years with overweight or obesity (body mass index [BMI] ≥ 23 kg/m*^2^*, Asian-specific cut-off) [30] and prediabetes, defined according to the American Diabetes Association (ADA) criteria as fasting blood glucose (FBG) 100–125 mg/dL and/or HbA1c 5.7–6.4% [31], confirmed on two separate occasions within 4 weeks prior to randomization. Additional requirements included stable body weight (±3 kg) during the preceding 3 months; insufficiently active (<150 min/wk of moderate-intensity physical activity and no structured resistance training (RT) > 1 session/wk during the prior 3 months); stable doses of medications affecting glucose, lipid, or blood pressure (BP) for at least 3 months; ability and willingness to comply with study procedures, including ingestion of study capsules and attendance at ≥85% of CBT sessions; adherence to protocol-specified caffeine and alcohol limits; and medical clearance for moderate-to-vigorous exercise (PAR-Q+ and physician approval). Exclusion criteria included a diagnosis of T2DM; current use of glucose-lowering medications or dietary supplements with metabolic effects; history of cardiovascular disease (CVD); uncontrolled hypertension (≥160/100 mmHg); clinically significant liver, kidney, or hematological disorders; pregnancy or lactation; allergy to gelatin or capsule ingredients; and any medical condition or contraindication that precluded safe participation in moderate-intensity physical activity.

#### 2.2.3. Randomization, Allocation, and Blinding

Participants were randomly assigned to one of four intervention arms: PLA, CBT, EATME, and CBT + EATME. Randomization was conducted after baseline assessments using a computer-generated minimization method stratified by age, sex, BMI, FBG, and HbA1c to ensure balanced allocation across groups. The randomization sequence was generated by an investigator not involved in participant recruitment, intervention delivery, or data collection. Allocation concealment was maintained using sequentially numbered, opaque, sealed envelopes prepared by an independent research assistant. Participant enrollment and group assignment were performed by a coordinator blinded to the randomization sequence. Supplementation codes were prepared and sealed in identical opaque envelopes by an independent researcher at the Kasetsart Agricultural and Agro-Industrial Product Improvement Institute, who also coded the capsules. These coded envelopes were provided to an assistant researcher responsible for capsule distribution. Trainers were aware of group allocation only for the exercise arms (CBT and CBT + EATME) to deliver the prescribed training. All participants were blinded to supplement assignment and to the overall study hypothesis. Outcome assessors and data analysts remained blinded to all group assignments until completion of the statistical analyses. This study employed a single-blind design in which participants, outcome assessors, and data analysts were blinded to supplement allocation and to the overall study hypothesis. Trainers were aware of group assignment only for the exercise arms (CBT and CBT + EATME) to supervise training sessions.

### 2.3. Experimental Procedures

This clinical trial comprised an 8-week intervention with comprehensive pre- and post-intervention assessments. Primary and secondary outcomes included anthropometry and body composition; glycemic markers (FBG, HbA1c, fasting insulin, HOMA-IR); inflammatory markers [high-sensitivity CRP (hs-CRP), IL-6, TNF-α]; adiponectin; lipid profiles and atherogenic indices; fitness measures; quality of life (QoL); lifestyle factors related to nutrition and physical activity; and clinical safety, including adverse event monitoring. All assessments were conducted at the Faculty of Sports and Health Science, Kasetsart University. Participants reported to the laboratory at 7:00 a.m. after a 12 h overnight fast and abstinence from alcohol, caffeine, and strenuous exercise. To minimize inter-observer variability, all anthropometric, body composition, and hemodynamic measurements were performed by the same trained research assistant. Testing was conducted at a consistent time of day (±1 h) under standardized laboratory conditions to control for diurnal variation.

**Figure 2:**
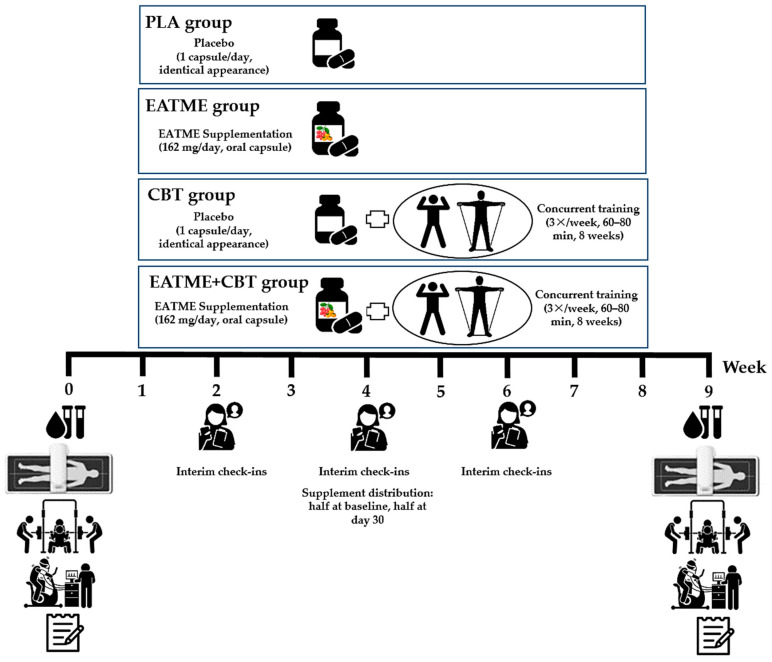
A schematic overview of the experimental procedures.

During the intervention, participants in the CBT and CBT + EATME groups performed supervised concurrent training three times per week in small groups. Each session consisted of moderate-to-vigorous aerobic training (AT) followed by RT using body weight and elastic bands, targeting all major muscle groups with progressive overload. Participants in all groups consumed one capsule daily after breakfast, corresponding to 162 mg/day of encapsulated polyphenol compounds or placebo. The microencapsulated polyphenol and placebo capsules were identical in size, color, and appearance, and were flavorless and odorless to maintain participant and investigator blinding throughout the study. Throughout the study, participants were instructed to maintain their habitual diet and usual physical activity outside the intervention and to abstain from additional vitamin or dietary supplements that could confound study outcomes. Adherence was monitored throughout the trial; participants were required to complete at least 80% of prescribed training sessions and supplement intake to be considered adherent. Participants were instructed to discontinue the supplement immediately and notify the investigators in the event of any adverse events. A schematic overview of the experimental procedures is shown in Figure 2.

### 2.4. Preparation of Supplement Extracts and Encapsulation

Dried and ground persimmon fruit (2 kg) and karonda fruit (2 kg) were separately subjected to ultrasound-assisted extraction. Each extraction employed 70% (*v*/*v*) ethanol as the solvent under optimized conditions: a solid-to-liquid ratio of 1:10 (*w*/*v*), ultrasound frequency of 19.8 kHz, power output of 20 kW, and an extraction duration of 3 h. Following extraction, each sample was filtered through Whatman No. 1 filter paper, and solvents were removed using a rotary evaporator at 45 °C under vacuum (0.08 mbar), yielding concentrated persimmon and karonda extracts. The final EATME formulation was prepared by combining the two extracts at a weight ratio of 70% persimmon extract and 30% karonda extract (*w*/*w*).

Microencapsulation of the formulated EATME extract was performed using maltodextrin as the encapsulating agent at an extract-to-maltodextrin ratio of 1:1.5. Briefly, 100 g of the EATME extract was dissolved in 400 mL of distilled water and sonicated in an ultrasonic water bath (Elma Schmidbauer GmbH, Singen, Germany) for 10 min at ambient temperature. The sonicated solution was gradually mixed with a maltodextrin solution under continuous stirring with a magnetic stirrer (C-MAG HS 7, IKA, Staufen, Germany) at 300 rpm for 60 min. The resulting mixture was further homogenized using a digital homogenizer (T18 Digital, IKA, Staufen, Germany) at 7000 rpm for 10 min. The homogenized mixture was then freeze-dried (Epsilon, Bangkok, Thailand) at −50 °C under vacuum (0.38–0.27 mbar) for 48 h. The freeze-dried material was ground into a fine powder using a mortar and encapsulated into gelatin capsules, each containing 162 mg of powder. High-performance liquid chromatography profiling identified several bioactive phenolic acids and flavonoids in the encapsulated powder, as summarized in the phytochemical characterization of the microencapsulated EATME extract in Supplementary Table S2.

### 2.5. Exercise Training Protocol

The concurrent training program was conducted three times per week for 8 weeks, with each supervised session lasting 60–80 min. Each session included a 10 min dynamic warm-up, 40–60 min of combined AT and RT, and a 10 min cool-down with static stretching. All sessions were delivered in small groups under the direct supervision of qualified sport scientists to ensure participant safety and protocol fidelity. AT involved low-impact dance performed at 70–80% of heart rate reserve and continuously monitored using heart rate sensors (H10, Polar Electro Inc., Kempele, Finland). Session duration was progressively increased from 20 min in weeks 1–2, to 25 min in weeks 3–4, and up to 30 min in weeks 5–8 to ensure progressive overload and maintain adherence.

RT targeted all major muscle groups using elastic bands and body weight. Exercises included chest press, seated row, lateral pulldown, leg press, leg curl, abdominal crunch, back extension, arm curl, arm extension, and squats. Participants performed two sets of 8–12 repetitions per exercise during weeks 1–4, progressing to three sets in weeks 5–8. Intensity was regulated using the OMNI-Resistance Exercise Scale (0 = extremely easy; 10 = extremely hard), corresponding to ~55–70% of one-repetition maximum (1RM) initially, and progressing to ~70–80% 1RM (OMNI-RES 7–8/10) during later weeks, provided correct technique was maintained [32]. Resistance was increased if participants could complete two additional repetitions in the final set while sustaining appropriate form. Participants were instructed on proper posture, movement control, and breathing to standardize technique. All exercises were performed in a controlled, self-paced manner. The program content was reviewed by independent strength training specialists and deemed appropriate for the study population.

### 2.6. Outcomes Measurement

#### 2.6.1. Anthropometry and Body Composition

Standing height was measured to the nearest 0.1 cm using a stadiometer (Health o Meter™, Sunbeam Products, Atlanta, GA, USA) with participants barefoot and aligned to the Frankfort plane. Body mass was assessed with a calibrated digital scale (Filizzola PL 150, Filizzola^®^ Ltd., São Paulo, Brazil), and BMI was calculated as weight divided by height squared (kg/m*^2^*). Body composition was assessed after an overnight fast using dual-energy X-ray absorptiometry (iDXA; GE Healthcare, Chicago, IL, USA). Parameters included body fat percentage (%BF), fat mass (FM), fat-free mass (FFM), lean mass (LM), and regional LM in the arms and legs. Central adiposity was evaluated by android and gynoid fat percentages and the android-to-gynoid (A/G) ratio. The scanner was calibrated daily, and all measurements were performed using standardized protocols to ensure accuracy and reproducibility.

#### 2.6.2. Blood Samples and Analysis

##### Blood Sampling

After a 12 h overnight fast and ≥72 h after the last training session, venous blood was drawn from the antecubital vein with participants resting supine for 10 min. Samples were collected sequentially into serum tubes (TNF-α, IL-6, hs-CRP, adiponectin, insulin, lipid profile, and liver/renal markers), EDTA tubes (HbA1c), and fluoride–oxalate tubes (plasma glucose). Serum was allowed to clot for 30 min before centrifugation at 1500× *g* for 10 min at 4 °C. EDTA samples were kept on ice and centrifuged within 60 min at 1500× *g* for 15 min at 4 °C. Plasma and serum aliquots were stored at −80 °C within 2 h. Hemolyzed samples were excluded. All assays were performed in duplicate, and each participant’s samples were analyzed within the same batch to minimize inter-assay variability.

##### Analysis of Glycemic Control Markers

FBG was measured using an automated chemistry analyzer (Thermo Fisher Scientific, Waltham, MA, USA) with the hexokinase method. Plasma from fluoride/oxalate tubes was processed within 1 h of collection and analyzed the same day or stored at 4 °C for ≤24 h; hemolyzed samples were excluded. Fasting insulin was assessed using an immunoassay analyzer (Siemens Healthcare Diagnostics, Mannheim, Germany), and insulin resistance was estimated using HOMA-IR, calculated as [fasting insulin (µIU/mL) × FBG (mmol/L)]/22.5 [33]. HbA1c (%) was determined from whole blood by immunoassay (Roche Cobas c502, Roche Diagnostics, Mannheim, Germany), with intra- and inter-assay coefficients of variation (CV) < 3%.

##### Analysis of Lipid Profiles and Atherogenic Indices

Serum total cholesterol (TC), triglycerides (TG), high-density lipoprotein cholesterol (HDL-c), and low-density lipoprotein cholesterol (LDL-c) were determined using an automated analyzer (Thermo Fisher Scientific Inc., Waltham, MA, USA). The atherogenic index of plasma (AIP) was calculated as log(TG/HDL-c), Castelli Risk Index I (CRI-I) as TC/HDL-c, Castelli Risk Index II (CRI-II) as LDL-c/HDL-c, and the atherogenic coefficient (AC) as (TC − HDL-c)/HDL-c.

##### Analysis of Inflammatory and Adipokine Markers

Serum concentrations of TNF-α, IL-6, and adiponectin were measured using commercially available ELISA kits (R&D Systems, Minneapolis, MN, USA) following the manufacturer’s instructions. Briefly, 96-well ELISA plates were coated with 100 μL of the respective capture antibody, sealed the plate, and incubated overnight at room temperature. Plates were then washed three times with wash buffer and blocked with reagent diluent for 1 h at room temperature. After three additional washes, 100 μL of either diluted serum samples or serially diluted standards were added to each well and incubated for 2 h at room temperature. Plates were washed again before adding 100 μL of detection antibody, followed by incubation for another 2 h at room temperature.

Subsequently, wells were washed and incubated with 100 μL of diluted streptavidin–HRP for 20 min at room temperature in the dark, then washed three times. Substrate solution (100 μL) was added to each well and incubated for 20 min at room temperature, after which the reaction was stopped with 50 μL of stop solution. Absorbance was measured at 450 nm with wavelength correction at 540 nm using a microplate reader (SpectraMax ABS, Molecular Devices LLC, San Jose, CA, USA). Cytokines and adiponectin concentrations were calculated from standard curves generated by serially diluted recombinant standards, with linear regression analysis (*R*^2^ ≥ 0.99). All assays were performed in duplicate, and intra- and inter-assay CVs were maintained at <10%.

Serum concentrations of hs-CRP were quantified using a particle-enhanced immunoturbidimetric assay on an automated clinical chemistry analyzer (Cobas c702, Roche Diagnostics, Mannheim, Germany), according to the manufacturer’s instructions. Each analytical run included calibrators and two-tier quality controls supplied by the manufacturer. The analytical sensitivity of the assay was 0.1 mg/L, and the intra- and inter-assay CVs were <5%. All samples were assayed in duplicate, and results are expressed in mg/L.

##### Analysis of Markers of Renal and Liver Functions

Serum biochemistry was analyzed using an automated chemistry analyzer (Cobas c502, Roche Diagnostics, Mannheim, Germany) with manufacturer-supplied reagents. BUN was measured by the urease–GLDH method, creatinine by an IDMS-traceable enzymatic assay, and eGFR calculated using the CKD-EPI 2021 equation [34]. Liver enzymes [(aspartate aminotransferase (AST), alanine aminotransferase (ALT), and alkaline phosphatase (ALP)] were determined by IFCC-recommended methods. All assays included internal quality control, with inter- and intra-assay CVs < 5%.

#### 2.6.3. Upper and Lower Muscular Strength

Muscular strength was assessed by estimating the 1RM for the leg press and bench press using the National Strength and Conditioning Association (NSCA) guidelines. Prior to testing, participants completed a standardized warm-up consisting of one set of 10 repetitions at a light load corresponding to ~12–15 RM. During testing, if participants were able to perform more than 10 repetitions, the load was increased incrementally by 30 pounds for the leg press and 10 pounds for the bench press. Each trial was separated by a 3 min rest interval to minimize fatigue. The 1RM was estimated based on the maximum load and repetitions completed using the NSCA prediction table. All assessments were supervised by an NSCA-certified strength and conditioning specialist to ensure safety and accuracy.

#### 2.6.4. Cardiorespiratory Fitness

Cardiorespiratory fitness was assessed using a multi-stage submaximal treadmill protocol with breath-by-breath gas analysis (Vmax^®^ Encore, CareFusion, Yorba Linda, CA, USA). Participants performed 2–4 continuous stages, each lasting 3 min, with workload progression corresponding to approximately 60–80% of age-predicted maximal heart rate. During each stage, steady-state oxygen uptake (*V̇*O_2_; 30–60 s average) and HR were recorded. Individual *V̇*O_2_–HR relationships were modeled using linear regression and extrapolated to age-predicted maximal heart rate [35] to estimate *V̇*O*_2_*max, in accordance with the American College of Sports Medicine guidelines [36].

#### 2.6.5. Quality of Life

QoL was assessed using the validated Thai version of the World Health Organization Quality of Life Brief (WHOQOL-BREF) questionnaire [37]. The instrument comprises 26 items covering four domains: physical health (7 items), psychological health (6 items), social relationships (3 items), and environment (8 items), along with two standalone items on overall QoL and general health. Items are rated on a 5-point Likert scale, with negatively phrased items reverse scored. Domain scores were calculated according to WHOQOL-BREF guidelines, with higher scores indicating better perceived QoL.

#### 2.6.6. Dietary Intake

Dietary intake was assessed at baseline and post-intervention using a 3-day food diary (two weekdays and one weekend day). Participants recorded all foods and beverages with the aid of a structured booklet and portion-size estimation tools. Dietitians reviewed diaries for accuracy and completeness. Energy and macronutrient intake were analyzed with INMUCAL-Nutrients V.3 software (Institute of Nutrition, Mahidol University, Nakhon Pathom, Thailand) [38].

### 2.7. Statistical Analysis

Normality was assessed using the Shapiro–Wilk test, and variables with non-normal distributions were log-transformed prior to analysis. Baseline differences among groups were examined using one-way ANOVA. To evaluate the combined effects of microencapsulated polyphenol supplementation and concurrent training, a two-way repeated-measures ANOVA [group (PLA, CBT, EATME, CBT + EATME) × time (baseline, 8 weeks)] was performed. When significant main effects or interactions were detected, post hoc pairwise comparisons were conducted using the Bonferroni correction to control for multiple testing. Analysis of covariance (ANCOVA) was additionally employed to compare post-intervention outcomes (HOMA-IR, TG, AC, and hs-CRP) among groups, with baseline values entered as covariates. Effect sizes were reported as partial *η*^2^ and interpreted as small (0.01), medium (0.06), or large (0.14). Statistical significance was set at *p* < 0.05. All analyses were performed using SPSS version 22.0 (SPSS Inc., Chicago, IL, USA).

## 3. Results

A total of forty-three adults completed the intervention and were included in the final analysis across four groups: PLA (*n* = 11), CBT (*n* = 10), EATME (*n* = 11), and combined CBT + EATME (*n* = 11). Participants were predominantly female (38 women and 5 men) with a mean age of 46.3 ± 9.0 years. Based on the Asian-specific BMI cut-off (≥23 kg/m^2^), the cohort was classified as overweight or obese, with a mean BMI of 26.1 ± 4.0 kg/m^2^.

All participants met the ADA criteria for prediabetes, with a mean FBG of 111.7 ± 17.2 mg/dL and HbA1c of 6.1 ± 0.7%. Resting blood pressure values were within the prehypertensive range (SBP: 124.2 ± 11.3 mmHg; DBP: 80.0 ± 8.5 mmHg). Physical activity assessments confirmed sedentary or insufficiently active status, with participants averaging 10.5 ± 0.5 h/day sitting, 5.5 ± 0.6 h/day walking, 1.0 ± 0.2 h/day in moderate activity, and 0.3 ± 0.3 h/day in vigorous activity. Dietary energy and macronutrient intake did not differ significantly within or between groups at any time point (Supplementary Table S3). No significant between-group differences were observed at baseline across demographic, anthropometric, metabolic, dietary, or activity variables (all *p* > 0.05; Table 1). Adherence to the intervention was excellent across all groups. The mean capsule intake compliance rates were 100% in the PLA and CBT groups, 99.6 ± 0.8% in the EATME group, and 99.2 ± 1.1% in the CBT + EATME group. Participants in the CBT and CBT + EATME arms completed 98.8 ± 2.1% and 98.4 ± 2.2% of the prescribed training sessions, respectively.

In terms of anthropometry and body composition, two-way ANOVA revealed significant main effects of time for body weight (*F*_(1,39)_ = 13.89, *p* = 0.001, *η^2^* = 0.263) and BMI (*F*_(1,39)_ = 12.75, *p* = 0.001, *η^2^* = 0.246), both representing large effect sizes. Reductions were observed in the CBT group (*p* < 0.01 and *p* < 0.05, respectively) and in the CBT + EATME group (*p* < 0.01 for both) compared with baseline, although no significant group × time interactions were detected. No significant changes were found for %BF, FM, FFM, LM, segmental LM (arms and legs), android or gynoid fat percentages, or the A/G ratio (all *p* > 0.05). These results are presented in Supplementary Table S4.

### 3.1. Metabolic Biomarkers

#### 3.1.1. Glycemic Control

**Figure 3:**
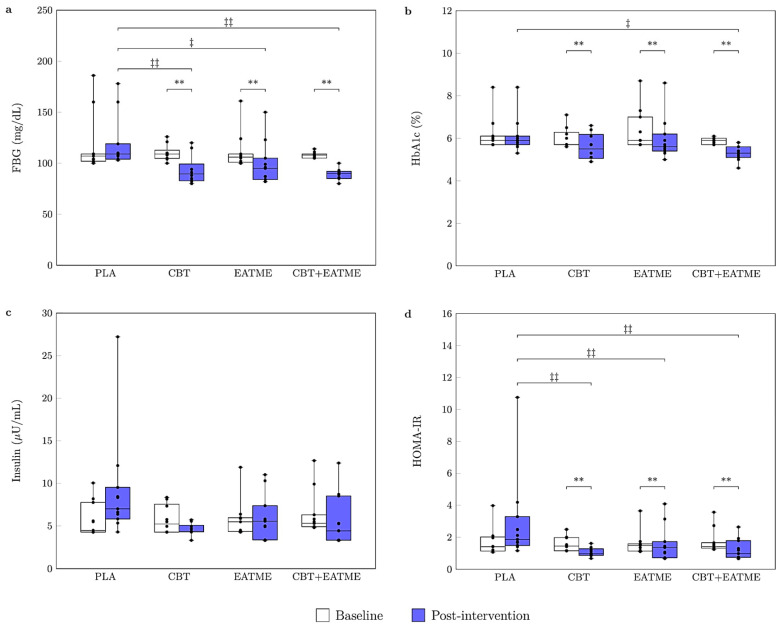
Boxplots for glycemic control parameters at baseline and after 8 weeks of intervention: (**a**) FBG; (**b**) HbA1c; (**c**) insulin; and (**d**) HOMA-IR. The upper, middle, and bottom horizontal lines of each box represent the 1st quartile, median, and 3rd quartile of the data, respectively, and the error bars indicate the minimum and maximum values. Abbreviations: PLA, placebo control group (*n* = 11); CBT, concurrent training group (*n* = 10); EATME, microencapsulated polyphenol compounds supplementation group (*n* = 11); CBT + EATME, combined concurrent training with microencapsulated polyphenol compounds supplementation group (*n* = 11); FBG, fasting blood glucose; HbA1c, glycated hemoglobin; HOMA-IR, homeostasis model assessment of insulin resistance. ** *p* < 0.01 vs. baseline within group; ^‡^ *p* < 0.05 and ^‡‡^ *p* < 0.01 vs. PLA.

A significant time effect was observed for FBG (*F*_(1,39)_ = 85.46, *p* < 0.001, *η^2^* = 0.687) and HbA1c (*F*_(1,39)_ = 39.55, *p* < 0.001, *η^2^* = 0.504), both indicating large effects. Significant group × time interactions were observed for FBG (*F*_(3,39)_ = 14.44, *p* < 0.001, *η^2^* = 0.526) and HbA1c (*F*_(3,39)_ = 4.69, *p* < 0.01, *η^2^* = 0.265). Post hoc analyses indicated significant reductions in FBG and HbA1c in the CBT (*p* < 0.01), EATME (*p* < 0.01), and CBT + EATME (*p* < 0.01) groups compared with baseline, whereas the PLA group showed no significant change. Between groups, CBT (*p* < 0.01), EATME (*p* < 0.05), and CBT + EATME (*p* < 0.01) produced greater reductions in FBG than PLA. For HbA1c, only CBT + EATME demonstrated a significantly greater reduction than PLA (*p* < 0.05). In contrast, fasting insulin did not change significantly over time and showed no group × time interaction. After adjusting for baseline values, ANCOVA revealed a significant group effect on post-intervention HOMA-IR (*F*_(3,38)_ = 14.78, *p* < 0.001, *η^2^* = 0.538), indicating differential improvements in insulin resistance across interventions. HOMA-IR levels were significantly reduced in the CBT (*p* < 0.01), EATME (*p* < 0.01), and CBT + EATME (*p* < 0.01) groups, each showing lower values compared with placebo (all *p* < 0.01). These findings are illustrated in Figure 3.

#### 3.1.2. Lipid Profiles and Atherogenic Indices

Two-way ANOVA revealed a significant main effect of time for LDL-C (*F*_(1,39)_ = 6.57, *p* < 0.05, *η^2^* = 0.144), with post hoc analysis showing a reduction in the CBT + EATME group (*p* < 0.05) compared with baseline; however, no significant group × time interaction was detected. In contrast, no significant time or interaction effects were observed for TC or HDL-C (all *p* > 0.05). Among the atherogenic indices, AIP demonstrated both a significant main effect of time (*F*_(1,39)_ = 4.17, *p* < 0.05, *η*^2^ = 0.097) and a significant group × time interaction (*F*_(3,39)_ = 7.32, *p* = 0.001, *η*^2^ = 0.360). Post hoc analyses indicated reductions in AIP in the CBT (*p* < 0.05) and EATME (*p* < 0.01) groups compared with baseline, while PLA exhibited a significant increase (*p* < 0.05); moreover, post-intervention AIP was lower in CBT compared with PLA (*p* < 0.05). CRI-II showed a significant main effect of time (*F*_(1,39)_ = 7.29, *p* = 0.010, *η*^2^ = 0.157), with post hoc analysis indicating a significant reduction in the CBT group (*p* < 0.05) compared with baseline; however, no significant group × time interaction was observed. By contrast, CRI-I showed no significant effects (all *p* > 0.05). After adjusting for baseline, ANCOVA revealed significant group effects for TG (*F*_(3,38)_ = 6.96, *p* = 0.001, *η*^2^ = 0.355) and AC (*F*_(3,38)_ = 3.25, *p* < 0.05, *η*^2^ = 0.204). TG decreased significantly in CBT (*p* < 0.05), EATME (*p* < 0.01), and CBT + EATME (*p* < 0.05). Post-intervention TG levels were lower in CBT (*p* < 0.05) and EATME (*p* < 0.01) compared with PLA, while the CBT + EATME group showed a non-significant trend toward lower values (*p* = 0.09; 95% CI: −90.6 to 4.1). AC was reduced in EATME compared with baseline (*p* < 0.05) and was also lower than PLA at post-intervention (*p* < 0.05). These findings are summarized in Table 2.

#### 3.1.3. Renal and Liver Functions

Renal and hepatic function markers remained largely unchanged, with no significant alterations in BUN, creatinine, eGFR, AST, or ALT (all *p* > 0.05). Although ALP increased significantly over time (*F*_(1,39)_ = 18.413, *p* < 0.001, *η*^2^ = 0.321), post hoc analyses indicated increases in the CBT (*p* < 0.05) and CBT + EATME (*p* < 0.05) groups compared with baseline, without a significant group × time interaction. Importantly, the observed ALP elevations remained within the normal physiological range, and no evidence of hepatic or renal impairment was detected. This mild increase may reflect enhanced bone turnover associated with the resistance and weight-bearing components of concurrent training, which stimulate osteoblastic activity and bone remodeling. Furthermore, no adverse events, allergic reactions, or harmful effects related to microencapsulated EATME extract or CBT were reported throughout the study. Detailed results are presented in Supplementary Table S5.

### 3.2. Inflammatory and Adipokine Markers

**Figure 4:**
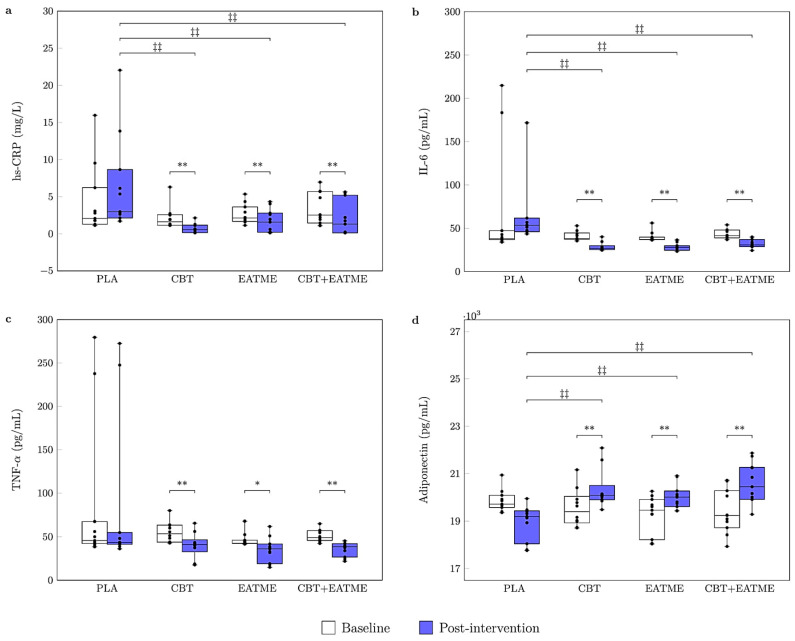
Boxplots for inflammatory and adipokine markers at baseline and after 8 weeks of intervention: (**a**) hs-CRP; (**b**) TNF-α; (**c**) IL-6; and (**d**) Adiponectin. The upper, middle, and bottom horizontal lines of each box represent the 1st quartile, median, and 3rd quartile of the data, respectively, and the error bars indicate the minimum and maximum values. Abbreviations: PLA, placebo control group (*n* = 11); CBT, concurrent training group (*n* = 10); EATME, microencapsulated polyphenol compounds supplementation group (*n* = 11); CBT + EATME, combined concurrent training with microencapsulated polyphenol compounds supplementation group (*n* = 11); hs-CRP, high-sensitive C-reactive protein; TNF-α, tumor necrosis factor-alpha; IL-6, interleukin-6. * *p* < 0.05 and ** *p* < 0.01 vs. baseline within group; ^‡‡^ *p* < 0.01 vs. PLA.

After adjusting for baseline values, ANCOVA revealed a significant group effect on post-intervention hs-CRP (*F*_(3,38)_ = 7.05, *p* = 0.001*, η*^2^ = 0.358). Significant reductions were observed in the CBT (*p* < 0.01), EATME (*p* < 0.01), and CBT + EATME (*p* < 0.01) groups, all of which showed lower hs-CRP levels compared with placebo (all *p* < 0.01). Two-way ANOVA demonstrated significant main effects of time for IL-6 (*F*(_1,39)_ = 20.94, *p* < 0.001, *η*^2^ = 0.349), TNF-α (*F*_(1,39)_ = 23.17, *p* < 0.001, *η*^2^ = 0.373), and adiponectin (*F*_(1,39)_ = 23.77, *p* < 0.001, *η*^2^ = 0.379). Significant group × time interactions were observed for IL-6 (*F*_(3,39)_ = 6.74, *p* = 0.001, *η*^2^ = 0.341) and adiponectin (*F*_(3,39)_ = 25.18, *p* < 0.001, partial *η*^2^ = 0.656), but not for TNF-α. Post hoc analyses indicated that IL-6 decreased significantly in the CBT (*p* < 0.01), EATME (*p* < 0.01), and CBT + EATME (*p* < 0.01) groups compared with baseline, with lower post-intervention levels than PLA (*p* < 0.01). TNF-α also decreased significantly in CBT (*p* < 0.01), EATME (*p* < 0.05), and CBT + EATME (*p* < 0.01), although no significant between-group differences were detected. By contrast, adiponectin increased significantly in CBT (*p* < 0.01), EATME (*p* < 0.01), and CBT + EATME (*p* < 0.01) compared with baseline, with all three intervention groups showing higher post-intervention values than PLA (*p* < 0.01). These results are illustrated in Figure 4.

### 3.3. Physical Fitness

For muscular strength, absolute bench press 1RM demonstrated significant main effects of time (*F*_(1,39)_ = 28.90, *p* < 0.001, *η*^2^ = 0.426) and group × time interaction (*F*_(3,39)_ = 3.25, *p* < 0.05, *η*^2^ = 0.200). Post hoc analyses showed significant improvements in the CBT (*p* < 0.01), EATME (*p* < 0.01), and CBT + EATME (*p* < 0.01) groups compared with baseline, with greater gains observed in CBT + EATME than PLA (*p* < 0.05). Relative bench press 1RM also revealed significant main effects of time (*F*_(1,39)_ = 30.08, *p* < 0.001, *η*^2^ = 0.435) and group × time interaction (*F*_(3,39)_ = 3.92, *p* < 0.05, *η*^2^ = 0.232), with increases in CBT (*p* < 0.01), EATME (*p* < 0.05), and CBT + EATME (*p* < 0.01); notably, CBT + EATME demonstrated greater improvements than PLA (*p* < 0.05). Absolute leg press 1RM improved significantly over time (*F_(_*_1,39*)*_ = 9.27, *p* < 0.01, *η*^2^ = 0.192), with within-group increases in CBT (*p* < 0.01) and CBT + EATME (*p* < 0.05), although no significant interaction was detected. Relative leg press 1RM showed significant main effects of time (*F*_(1,39_) = 18.01, *p* < 0.001, *η*^2^ = 0.316) and group × time interaction (*F*_(3,39)_ = 4.36, *p* = 0.010, *η*^2^ = 0.251), with significant increases in CBT (*p* < 0.01) and CBT + EATME (*p* < 0.01), both of which exceeded the improvements observed in PLA (*p* < 0.05). These results are presented in Table 3. For cardiorespiratory fitness, *V̇*O_2_max demonstrated significant main effects of time (*F*(_1,39)_ = 11.54, *p* < 0.01, *η*^2^ = 0.228) and group × time interaction (*F*_(3,39)_ = 3.66, *p* < 0.05, *η*^2^ = 0.219). Post hoc analyses revealed significant improvements in CBT (*p* < 0.01) and CBT + EATME (*p* < 0.01) compared with baseline, with both groups exhibiting greater gains than PLA (*p* < 0.05). These results are presented in Table 3.

### 3.4. Quality of Life

Two-way ANOVA demonstrated significant main effects of time for the physical domain (*F*_(1,39)_ = 13.98, *p* = 0.001, *η*^2^ = 0.264), psychological domain (*F*_(1,39)_ = 4.59, *p* < 0.05, *η*^2^ = 0.105), environment (*F*_(1,39)_ = 5.24, *p* < 0.05, *η*^2^ = 0.118), and overall QoL (*F*_(1,39)_ = 22.50, *p* < 0.001, *η*^2^ = 0.366), but not for the social domain. Significant group × time interactions were observed for the psychological domain (*F*_(3,39)_ = 3.08, *p* < 0.05, *η*^2^ = 0.192) and overall QoL (*F*_(3,39)_ = 5.81, *p* < 0.01, *η*^2^ = 0.309). Post hoc analyses revealed that physical domain scores improved significantly in CBT (*p* < 0.01) and CBT + EATME (*p* < 0.01) compared with baseline. Psychological domain scores improved significantly in CBT + EATME (*p* < 0.01) compared with baseline and were higher than PLA at post-intervention (*p* < 0.05). Environment scores increased significantly in CBT (*p* < 0.01) and CBT + EATME (*p* < 0.05) compared with baseline. Overall QoL improved significantly in CBT (*p* < 0.01), EATME (*p* < 0.05), and CBT + EATME (*p* < 0.01) compared with baseline, with greater improvements observed in CBT (*p* < 0.05) compared with PLA. No significant changes were observed in the social domain across time or between groups. These findings are summarized in Supplementary Table S6.

## 4. Discussion

This pilot randomized controlled trial demonstrated that concurrent training and supplementation with a microencapsulated persimmon–karonda polyphenol formulation, administered individually or in combination, produced significant benefits in adults with prediabetes and overweight. Improvements were observed in glycemic control, systemic inflammation, adiponectin concentrations, lipid profiles, and atherogenic indices, accompanied by gains in muscular strength, cardiorespiratory fitness, and QoL. These findings are consistent with prior evidence that lifestyle-based interventions can improve metabolic health in at-risk populations, but extend current knowledge by showing that structured exercise combined with bioavailable polyphenols yields broader and more clinically meaningful effects. Importantly, the interventions were well tolerated, with no adverse events or evidence of hepatic or renal toxicity, supporting their safety and feasibility for use in this population.

The reductions in FBG (11–18%), HbA1c (6–10%), and HOMAIR (15–27%) across intervention groups represent clinically meaningful improvements in glycemic control within a relatively short 8-week period. The marked increase in the proportion of participants achieving normoglycemia—with 72–82% attaining FBG < 100 mg/dL and up to 73% reaching HbA1c < 5.7%—underscores the clinical relevance of these findings beyond statistical significance. Although fasting insulin did not change significantly, the 27.9% reduction in HOMA-IR in the combined group indicates a favorable trend toward enhanced insulin sensitivity, which may become significant in longer trials or larger cohorts. Collectively, these findings demonstrate that both concurrent training and microencapsulated polyphenol supplementation improved glycemic regulation, with the combined intervention eliciting the most robust benefits.

These findings are consistent with previous research demonstrating that AT and RT support glycemic regulation through distinct yet complementary mechanisms. AT is particularly effective in improving glucose uptake and enhancing metabolic flexibility [39,40], whereas RT enhances skeletal muscle mass and glucose storage capacity, as demonstrated in prediabetic obese me [41], middle-aged individuals with morbid obesity and T2DM [42], and older adults with obesity and/or T2DM [39]. When combined as concurrent training, these adaptations reinforce one another, leading to greater improvements in insulin sensitivity. In parallel, polyphenols such as quercetin, ferulic acid, and rutin have been associated with improved glycemic regulation and reduced hepatic glucose output [43,44,45]. The convergence of these diet-induced adaptations with exercise-related improvements in glucose uptake and mitochondrial function may help explain the superior glycemic outcomes observed with the combined intervention. 

Notably, previous trials with non-encapsulated polyphenols such as resveratrol, quercetin, or berry extracts often reported neutral or inconsistent effects on glycemic and inflammatory markers, largely due to poor stability and limited bioavailability [46,47,48]. In contrast, encapsulated formulations—including micro- and nano-based delivery systems—improve compound stability and systemic exposure, and have shown more consistent benefits on FBG, insulin resistance, and lipid profiles [49,50,51]. These advances provide a strong mechanistic rationale for the present findings, in which microencapsulated persimmon–karonda supplementation, particularly when combined with concurrent training, produced clinically meaningful effects beyond those typically expected from polyphenols alone.

Beyond metabolic outcomes, both concurrent training and polyphenol supplementation modulated inflammatory pathways and increased adiponectin, providing an additional mechanistic link to improved insulin sensitivity. Chronic low-grade inflammation is a hallmark of prediabetes and obesity, typically characterized by elevated levels of hs-CRP, IL-6, and TNF-α [52]. In this trial, all active interventions reduced systemic inflammation compared with placebo, with significant declines in hs-CRP and consistent reductions in IL-6 across the CBT, EATME, and CBT + EATME groups. TNF-α also decreased within intervention groups, although between-group differences were less pronounced. Together, these findings highlight the complementary effects of exercise and bioavailable polyphenols in attenuating inflammation and enhancing adipokine regulation, thereby contributing to improved metabolic health.

The synergistic effects of concurrent training and polyphenol supplementation may be mediated through convergent molecular pathways that regulate energy metabolism and inflammation. Exercise-induced activation of AMP-activated protein kinase (AMPK) promotes glucose uptake, fatty acid oxidation, and mitochondrial biogenesis while concurrently suppressing lipogenesis. Polyphenols further potentiate AMPK signaling and inhibit nuclear factor κB–mediated proinflammatory cascades, thereby reducing the expression of cytokines and inflammatory mediators such as TNF-α, IL-6, and hs-CRP [7,8,53,54]. Moreover, both exercise and polyphenols upregulate peroxisome proliferator-activated receptor gamma and adiponectin expression, supporting enhanced insulin sensitivity and lipid metabolism [55,56].

These anti-inflammatory adaptations were accompanied by substantial increases in adiponectin across all active interventions, with levels consistently higher than those observed in the placebo group. Adiponectin is widely recognized as a key regulator of insulin sensitivity, facilitating glucose utilization and lipid metabolism [5]. In the present trial, however, no direct correlations were observed between adiponectin and inflammatory markers, suggesting that their improvements may reflect parallel yet independent pathways contributing to enhanced insulin sensitivity. Nevertheless, the consistent elevation of adiponectin across intervention groups indicates that it may still exert an indirect but supportive role in promoting overall metabolic health.

Notably, only in the CBT + EATME group did reductions in hs-CRP correlate positively with improvements in HOMA-IR (*r* = 0.627, *p* < 0.05), indicating that suppression of inflammation translated into better glycemic control under the combined intervention. This observation suggests that improvements in glycemic regulation may be partly mediated by attenuation of systemic inflammation, offering novel insight into the synergistic effects of exercise and polyphenol supplementation. Collectively, these results demonstrate that concurrent training and microencapsulated polyphenol supplementation, particularly in combination, exert clinically meaningful benefits on systemic inflammation and adipokine regulation compared with placebo.

The improvements observed in TG and atherogenic indices provide an additional link to reduced cardiometabolic risk. Elevated TG and unfavorable indices such as AIP and AC are well established as predictors of insulin resistance and CVD [57]. In this trial, reductions in TG across all active interventions, together with lower AIP in CBT and EATME groups and decreased AC in EATME group, indicate a shift toward a less atherogenic lipid profile that is likely to contribute to improved metabolic health. Prior studies have shown that polyphenols, including quercetin, ferulic acid, and rutin, are associated with improved lipid metabolism and lower TG concentrations [58,59]. Moreover, encapsulated formulations, such as nano-micelle curcumin, have demonstrated more consistent reductions in TG in patients with T2DM and MetS [49,50,60], highlighting the role of delivery technologies in enhancing the efficacy of polyphenols. concurrent training, particularly AT, has also been linked to increased TG clearance and improved lipid handling [61]. Collectively, these complementary adaptations provide a coherent explanation for the lipid-lowering effects observed in this study.

Despite significant reductions in body weight and BMI in the CBT and CBT + EATME groups, no corresponding changes were observed in %BF, FM, or LM. These findings suggest that improvements in glycemic regulation and cardiometabolic risk are unlikely to be driven by major alterations in body composition during the relatively short 8-week intervention. Instead, functional adaptations in glucose metabolism, inflammation, and fitness appear to play a more central role. This interpretation is consistent with prior studies reporting that exercise and polyphenol supplementation improve insulin sensitivity and inflammatory profiles independently of substantial changes in adiposity [62,63]. The non-significant group-by-time interactions underscore that the modest reductions in body weight and BMI were not the principal mediators of the metabolic benefits achieved.

Muscular strength improved in both upper and lower body, with the largest gains observed in CBT + EATME compared with PLA. Cardiorespiratory fitness, as reflected by *V̇*O_2_max, also increased significantly in CBT and CBT + EATME groups, both outperforming placebo. QoL improved across several domains, with psychological well-being enhanced only in CBT + EATME group and overall scores highest in CBT group. The superior benefits of the combined intervention are plausibly explained by complementary mechanisms: aerobic and resistance components of concurrent training promote neuromuscular adaptation and mitochondrial biogenesis [64,65], while polyphenols reduce inflammation and oxidative stress, thereby supporting recovery [54] and amplifying training-induced adaptations. Clinically, gains in strength and aerobic capacity are strongly associated with reduced incidence of T2DM and all-cause mortality [66].

Improvements in perceived QoL and psychological well-being are known to reduce the risks of depression, frailty, and functional decline [67,68]. In the present study, such benefits were evident in the CBT group for overall QoL and in the CBT + EATME group for psychological well-being, both showing large effect sizes indicative of clinically meaningful change. These improvements likely reflect the combined physiological and psychosocial effects of concurrent training—such as enhanced mood, self-efficacy, and stress regulation [69]—together with the neuroprotective and mood-modulating properties of polyphenol supplementation, which may involve dopaminergic and serotonergic signaling and upregulation of brain-derived neurotrophic factor [70]. Collectively, these findings suggest that the psychological benefits observed are not only statistically significant but also clinically relevant, underscoring the potential of integrated exercise–nutraceutical strategies to enhance overall well-being in individuals with prediabetes.

This study has several limitations. The small sample size and short intervention duration limit both the generalizability and statistical power of the findings. The sample size was estimated based on a relatively large effect size from a previous nutraceutical trial, which may have led to an overestimation of statistical power; thus, the present findings should be regarded as exploratory and interpreted with caution. The a priori effect size was likely optimistic, indicating that the study was adequately powered to detect medium-to-large effects but underpowered for small effects and multiple secondary outcomes. The observed reductions in glycemic indices may partly reflect within-group changes, and some degree of regression to the mean cannot be excluded. Although Bonferroni corrections were applied for post hoc comparisons, the large number of endpoints tested may still increase the likelihood of type I error, particularly for secondary outcomes.

Blinding could not be implemented for the exercise component, as participants and trainers in the CBT and CBT + EATME groups were aware of group allocation, which may have introduced performance bias. Moreover, the majority of participants were female (approximately 88%), which may have influenced physiological and hormonal responses to exercise and supplementation. This gender imbalance further limits the generalizability of the findings; therefore, future studies should recruit more balanced cohorts to clarify potential sex-specific adaptations and responses.

Participants were instructed to maintain their habitual diet and physical activity throughout the intervention; however, compliance was assessed using 3-day food diaries and self-reported activity logs, which are prone to recall bias and underreporting. Consequently, residual confounding from uncontrolled dietary and activity factors cannot be ruled out. As a single-center trial, replication in larger and more diverse populations with longer intervention and follow-up periods is warranted to confirm the durability and scalability of these effects. Finally, although microencapsulation enhances polyphenol stability and bioavailability, circulating or urinary polyphenol metabolites and total antioxidant capacity were not measured. Future trials incorporating such biomarkers are needed to verify absorption and elucidate mechanistic links between polyphenol bioavailability and metabolic outcomes.

## 5. Conclusions

As an exploratory pilot randomized controlled trial, this study demonstrated that concurrent training combined with a microencapsulated persimmon–karonda polyphenol formulation significantly improved glycemic control, lipid profiles, inflammatory markers, adiponectin, muscular strength, cardiorespiratory fitness, and QoL in adults with prediabetes and overweight. The combined intervention produced the most consistent and pronounced benefits, underscoring the complementary effects of structured exercise and polyphenol supplementation. These findings support the feasibility and potential efficacy of integrated lifestyle–nutraceutical strategies as practical and sustainable approaches to reduce diabetes risk and promote overall health in at-risk populations, thereby contributing to the growing body of evidence on prediabetes management.

## Figures and Tables

**Table 1:** Baseline characteristics of the participants.

	Total (*n* = 43)	PLA (*n* = 11)	CBT (*n* = 10)	EATME (*n* = 11)	CBT + EATME (*n* = 11)	*F*	*p*-Value
Age (yr)	46.3 ± 9.0	44.4 ± 6.4	48.7 ± 11.1	47.9 ± 7.3	44.3 ± 11.0	0.689	0.564
Gender (F/M)	38/5	9/2	9/1	10/1	10/1		0.891
Height (cm)	159.0 ± 8.2	160.3 ± 9.3	158.0 ± 8.5	159.5 ± 7.9	158.3 ± 8.2	0.172	0.914
Weight (kg)	66.0 ± 11.3	68.5 ± 14.2	64.9 ± 10.3	67.0 ± 8.4	63.4 ± 12.1	0.417	0.742
BMI (kg/m^2^)	26.1 ± 4.0	26.5 ± 4.1	26.0 ± 3.3	26.5 ± 3.8	25.4 ± 5.0	0.178	0.911
FBG (mg/dL)	111.7 ± 17.2	117.4 ± 28.3	110.1 ± 7.8	111.1 ± 17.9	107.9 ± 2.8	0.594	0.622
HbA1c (%)	6.1 ± 0.7	6.2 ± 0.8	6.0 ± 0.5	6.3 ± 1.0	5.9 ± 0.1	0.953	0.425
Resting SBP (mmHg)	124.2 ± 11.3	127.4 ± 17.6	123.8 ± 11.7	123.9 ± 9.0	121.7 ± 4.5	0.450	0.719
Resting DBP (mmHg)	80.0 ± 8.5	79.6 ± 10.8	77.5 ± 6.3	82.6 ± 8.0	80.1 ± 8.5	0.636	0.596
Resting MAP (mmHg)	94.7 ± 8.4	95.3 ± 12.1	92.8 ± 7.5	96.5 ± 7.2	93.9 ± 6.6	0.360	0.782
Resting HR (beats/min)	71.4 ± 10.8	67.6 ± 7.5	69.7 ± 11.6	72.6 ± 11.4	75.6 ± 11.9	1.149	0.341
Physical activity levels							
Sitting (h/day)	10.5 ± 0.5	10.4 ± 0.5	10.5 ± 0.6	10.6 ± 0.5	10.4 ± 0.6	0.357	0.785
Walking (h/day)	5.5 ± 0.6	5.6 ± 0.5	5.4 ± 0.6	5.4 ± 0.6	5.6 ± 0.6	0.530	0.664
Moderate activity (h/day)	1.0 ± 0.2	1.1 ± 0.2	1.1 ± 0.2	1.0 ± 0.2	1.0 ± 0.0	0.591	0.625
Vigorous activity (h/day)	0.3 ± 0.3	0.3 ± 0.3	0.3 ± 0.2	0.2 ± 0.3	0.4 ± 0.2	0.347	0.791

Data are expressed as mean ± SD and frequency (*n*). Abbreviations: PLA, placebo control group; CBT, concurrent training group; EATME, microencapsulated polyphenol compounds supplementation group; CBT + EATME, combined concurrent training with microencapsulated polyphenol compounds supplementation group; BMI, body mass index; FBG, fasting blood glucose; HbA1c, glycated hemoglobin; SBP, systolic blood pressure; DBP, diastolic blood pressure; MAP, mean arterial pressure; HR, heart rate.

**Table 2:** Lipid profiles and atherogenic indices at baseline and after 8 weeks of intervention.

	PLA (*n* = 11)		CBT (*n* = 10)		EATME (*n* = 11)		CBT + EATME (*n* = 11)		Time Effect *η*^2^ (*p*-Value)	Group × Time Interaction *η*^2^ (*p*-Value)
	Baseline	Post-Test	Baseline	Post-Test	Baseline	Post-Test	Baseline	Post-Test		
TC (mg/dL)	212.4 ± 42.3	211.9 ± 43.6	207.5 ± 33.5	200.1 ± 32.6	207.2 ± 19.8	211.6 ± 21.4	205.6 ± 28.8	208.3 ± 33.3	0.000 (0.957)	0.049 (0.573)
TG (mg/dL)	131.6 ± 40.2	167.8 ± 83.8 ^a^**	116.0 ± 30.2	97.7 ± 22.9 ^a^*^,b^*	145.4 ± 51.9	106.2 ± 40.7 ^a^**^,b^**	114.5 ± 36.4	109.8 ± 39.5 ^a^*		0.366 (0.001) ^††^
HDL-c (mg/dL)	55.4 ± 12.7	52.1 ± 11.5	59.2 ± 12.5	61.3 ± 10.0	51.5 ± 11.9	55.5 ± 11.6	62.5 ± 10.1	62.8 ± 12.9	0.014 (0.466)	0.134 (0.129)
LDL-c (mg/dL)	146.4 ± 37.1	136.6 ± 32.8	138.5 ± 30.4	128.2 ± 23.9	138.5 ± 20.5	137.1 ± 20.2	138.3 ± 21.4	126.4 ± 27.0 ^a^*	0.144 (0.014) ^†^	0.040 (0.657)
AIP	0.4 ± 0.2	0.5 ± 0.3 ^a^*	0.3 ± 0.1	0.2 ± 0.2 ^a^*^,b^*	0.4 ± 0.3	0.3 ± 0.2 ^a^**	0.3 ± 0.2	0.2 ± 0.2	0.097 (0.048) ^†^	0.360 (0.001) ^††^
CRI-I	4.0 ± 1.1	4.2 ± 1.0	3.6 ± 0.8	3.3 ± 0.6	4.2 ± 0.9	4.0 ± 0.9	3.4 ± 0.7	3.4 ± 0.7	0.020 (0.372)	0.136 (0.124)
CRI-II	2.8 ± 0.9	2.7 ± 0.8	2.4 ± 0.7	2.1 ± 0.5 ^a^*	2.8 ± 0.8	2.6 ± 0.7	2.3 ± 0.5	2.1 ± 0.6	0.157 (0.010) ^††^	0.042 (0.636)
AC	3.0 ± 1.1	3.2 ± 1.2	2.6 ± 0.8	2.5 ± 0.8	3.2 ± 0.9	2.9 ± 0.7 ^a^*^,b^*	2.4 ± 0.7	2.4 ± 0.8		0.204 (0.032) ^††^

Data are expressed as mean ± SD. Abbreviations: PLA, placebo control group; CBT, concurrent training group; EATME, microencapsulated polyphenol compounds supplementation group; CBT + EATME, combined concurrent training with microencapsulated polyphenol compounds supplementation group; TC, total cholesterol; TG, triglyceride; HDL-c, high-density lipoprotein cholesterol; LDL-c, low-density lipoprotein cholesterol; AIP, atherogenic index of plasma; CRI-I, Castelli Risk Index I; CRI-II, Castelli Risk Index II; AC, atherogenic coefficient. ^†^ denotes a small-to-moderate effect size (0.06 ≤ partial *η*^2^ < 0.14); ^††^ denotes a large effect size (partial *η*^2^ ≥ 0.14). * and ** indicate statistical significance at *p* < 0.05 and *p* < 0.01, respectively. Superscript a indicates a significant within-group difference from baseline; superscript b indicates a significant difference from the PLA group after 8 weeks of intervention.

**Table 3:** Physical fitness parameters at baseline and after 8 weeks of intervention.

	PLA (*n* = 11)		CBT (*n* = 10)		EATME (*n* = 11)		CBT + EATME (*n* = 11)		Time Effect *η*^2^ (*p*-Value)	Group × Time Interaction *η*^2^ (*p*-Value)
	Baseline	Post-Test	Baseline	Post-Test	Baseline	Post-Test	Baseline	Post-Test		
Absolute bench press 1RM (kg)	34.4 ± 14.8	34.6 ± 13.6	33.5 ± 9.5	37.0 ± 10.5 ^a^**	35.8 ± 10.8	38.6 ± 12.2 ^a^**	31.1 ± 7.1	35.3 ± 5.6 ^a^**^,b^*	0.426 (0.000) ^††^	0.200 (0.032) ^††^
**Relative** bench press 1RM (kg/BW)	0.5 ± 0.2	0.5 ± 0.1	0.5 ± 0.1	0.6 ±0.2 ^a^**	0.5 ± 0.2	0.6 ± 0.2 ^a^*	0.5 ± 0.1	0.6 ± 0.1 ^a^**^,b^*	0.435 (0.000) ^††^	0.232 (0.015) ^††^
Absolute leg press 1RM (kg)	226.6 ± 76.6	223.6 ± 79.5	201.2 ± 43.8	220.1 ± 55.9 ^a^**	212.5 ± 56.4	219.6 ± 66.2	198.2 ± 59.4	210.0 ± 61.7 ^a^*	0.192 (0.004) ^††^	0.163 (0.072)
**Relative** leg press 1RM (kg/BW)	3.3 ± 0.7	3.2 ± 0.7	3.1 ± 0.4	3.5 ± 0.5 ^a^**^,b^*	3.2 ± 0.9	3.4 ± 1.0	3.1 ± 0.5	3.4 ± 0.3 ^a^**^,b^*	0.316 (0.000) ^††^	0.251 (0.010) ^††^
*V̇*O_2_max (mL/kg/min)	36.7 ± 4.7	36.7 ± 4.6	35.4 ± 3.9	39.0 ± 4.3 ^a^**^,b^*	35.9 ± 4.5	36.0 ± 5.0	35.7 ± 5.5	38.4 ± 3.8 ^a^**^,b^*	0.228 (0.002) ^††^	0.219 (0.021) ^††^

Data are expressed as mean ± SD. Abbreviations: PLA, placebo control group; CBT, concurrent training group; EATME, microencapsulated polyphenol compounds supplementation group; CBT + EATME, combined concurrent training with microencapsulated polyphenol compounds supplementation group; 1RM, one repetition maximum; *V̇*O_2_max, maximum oxygen consumption. ^††^ denotes a large effect size (partial *η*^2^ ≥ 0.14). * and ** indicate statistical significance at *p* < 0.05 and *p* < 0.01, respectively. Superscript a indicates a significant within-group difference from baseline; superscript b indicates a significant difference from the PLA group after 8 weeks of intervention.

## Data Availability

The original contributions presented in this study are included in the article and its Supplementary Material. Further inquiries can be directed to the corresponding author.

## Supplementary Materials

The following supporting information can be downloaded at https://www.mdpi.com/article/10.3390/nu17213358/s1, Table S1: Consort 2025 checklist of this study; Table S2: Phytochemical characterization of microencapsulated persimmon (*Diospyros kaki* L.) and karonda (*Carissa carandas* L.) extracts; Table S3: Dietary energy and macronutrient intake of participants in each group before and after the intervention; Table S4: Anthropometry and body composition parameters at baseline and after 8 weeks of intervention; Table S5: Renal and hepatic function markers at baseline and after 8 weeks of intervention; Table S6: Changes in quality of life parameters at baseline and after 8 weeks of intervention.

## Abbreviations

The following abbreviations are used in this manuscript:

T2DM	Type 2 diabetes mellitus
CBT	Concurrent training group
EATME	Microencapsulated polyphenol compounds supplementation group
CBT + EATME	Combined concurrent training with microencapsulated polyphenol compounds supplementation group
PLA	Placebo group
FBG	Fasting blood glucose
HbA1c	Glycated hemoglobin
HOMA-IR	Homeostasis model assessment of insulin resistance
hs-CRP	High-sensitive C-reactive protein
TNF-α	Tumor necrosis factor-alpha
IL-6	Iinterleukin-6
CRP	C-reactive protein
QoL	Quality of life
MetS	Metabolic syndrome
*V̇*O_2_max	Maximum oxygen consumption
HR	Heart rate
SBP	Systolic blood pressure
DBP	Diastolic blood pressure
MAP	Mean arterial pressure
1RM	One-repetition maximum
CVD	Cardiovascular disease
AT	Aerobic training
RT	Resistance training
%BF	Body fat percentage
FM	Fat mass
FFM	Fat-free mass
LM	Lean mass
A/G ratio	Android and gynoid fat ratio
CV	Coefficients of variation
TC	Total cholesterol
TG	Triglycerides
HDL-c	High-density lipoprotein cholesterol
LDL-c	Low-density lipoprotein cholesterol
AIP	Atherogenic index of plasma
CRI-I	Castelli Risk Index I
CRI-II	Castelli Risk Index II
AC	Atherogenic coefficient
BUN	Blood urea nitrogen
eGFR	Estimated glomerular filtration rate
AST	Aspartate aminotransferase
ALT	Alanine aminotransferase
ALP	Alkaline phosphatase
NSCA	National Strength and Conditioning Association
WHOQOL-BREF	World Health Organization quality of life brief questionnaire
