# Supplementary material for: Synergistic Effects of Microencapsulated Polyphenols and Concurrent Training on Metabolic Health and Fitness in Overweight/Obese Adults with Prediabetes
Source: Nutrients. 2025 Oct 25;17(21):3358. doi: 10.3390/nu17213358 (PMC12608511; doi:10.3390/nu17213358)
Supplement: Supplementary file 1 [file nutrients-17-03358-s001.zip › nutrients-3926618-supplementary.pdf]

Table S1: Consort 2025 checklist of this study

Section/topic	No	CONSORT 2025 checklist item description	Reported on page no.
**Title and abstract**			
Title and structured abstract	1a	Identification as a randomised trial	1
	1b	Structured summary of the trial design, methods, results, and conclusions	1
**Open science**			
Trial registration	2	Name of trial registry, identifying number (with URL) and date of registration	1, 3, and 20
Protocol and statistical analysis plan	3	Where the trial protocol and statistical analysis plan can be accessed	20
Data sharing	4	Where and how the individual de-identified participant data (including data dictionary), statistical code and any other materials can be accessed	20
Funding and conflicts of interest	5a	Sources of funding and other support (eg, supply of drugs), and role of funders in the design, conduct, analysis and reporting of the trial	19
	5b	Financial and other conflicts of interest of the manuscript authors	19
**Introduction**			
Background and rationale	6	Scientific background and rationale	2-3
Objectives	7	Specific objectives related to benefits and harms	3
**Methods**			
Patient and public involvement	8	Details of patient or public involvement in the design, conduct and reporting of the trial	3-4#
Trial design	9	Description of trial design including type of trial (eg, parallel group, crossover), allocation ratio, and framework (eg, superiority, equivalence, non-inferiority, exploratory)	3-4
Changes to trial protocol	10	Important changes to the trial after it commenced including any outcomes or analyses that were not prespecified, with reason	N/A
Trial setting	11	Settings (eg, community, hospital) and locations (eg, countries, sites) where the trial was conducted	3
Eligibility criteria	12a	Eligibility criteria for participants	4
	12b	If applicable, eligibility criteria for sites and for individuals delivering the interventions (eg, surgeons, physiotherapists)	5-6
Intervention and comparator	13	Intervention and comparator with sufficient details to allow replication. If relevant, where additional materials describing the intervention and comparator (eg, intervention manual) can be accessed	5-7

Section/topic	No	CONSORT 2025 checklist item description	Reported on page no.
Outcomes	14	Prespecified primary and secondary outcomes, including the specific measurement variable (eg, systolic blood pressure), analysis metric (eg, change from baseline, final value, time to event), method of aggregation (eg, median, proportion), and time point for each outcome	7-9
Harms	15	How harms were defined and assessed (eg, systematically, non-systematically)	13 and 16
Sample size	16a	How sample size was determined, including all assumptions supporting the sample size calculation	4
	16b	Explanation of any interim analyses and stopping guidelines	5-6
Randomisation:			
Sequence generation	17a	Who generated the random allocation sequence and the method used	5
	17b	Type of randomisation and details of any restriction (eg, stratification, blocking and block size)	5♯
Allocation concealment mechanism	18	Mechanism used to implement the random allocation sequence (eg, central computer/telephone; sequentially numbered, opaque, sealed containers), describing any steps to conceal the sequence until interventions were assigned	5
Implementation	19	Whether the personnel who enrolled and those who assigned participants to the interventions had access to the random allocation sequence	5
Blinding	20a	Who was blinded after assignment to interventions (eg, participants, care providers, outcome assessors, data analysts)	5
	20b	If blinded, how blinding was achieved and description of the similarity of interventions	5
Statistical methods	21a	Statistical methods used to compare groups for primary and secondary outcomes, including harms	10
	21b	Definition of who is included in each analysis (eg, all randomised participants), and in which group	10
	21c	How missing data were handled in the analysis	3
	21d	Methods for any additional analyses (eg, subgroup and sensitivity analyses), distinguishing prespecified from post hoc	10
**Results**			
Participant flow, including flow diagram	22a	For each group, the numbers of participants who were randomly assigned, received intended intervention, and were analysed for the primary outcome	3-4
	22b	For each group, losses and exclusions after randomisation, together with reasons	3-4
Recruitment	23a	Dates defining the periods of recruitment and follow-up for outcomes of benefits and harms	3-4
	23b	If relevant, why the trial ended or was stopped	N/A
Intervention and comparator delivery	24a	Intervention and comparator as they were actually administered (eg, where appropriate, who delivered the intervention/comparator, how participants adhered, whether they were delivered as intended (fidelity)	5-6

Section/topic	No	CONSORT 2025 checklist item description	Reported on page no.
	24b	Concomitant care received during the trial for each group	6-7
Baseline data	25	A table showing baseline demographic and clinical characteristics for each group	10
Numbers analysed, outcomes and estimation	26	For each primary and secondary outcome, by group:	
		● the number of participants included in the analysis	4 and 10
		● the number of participants with available data at the outcome time point	4 and 10
		● result for each group, and the estimated effect size and its precision (such as 95% confidence interval)	10-16
		● for binary outcomes, presentation of both absolute and relative effect size	10-16
Harms	27	All harms or unintended events in each group	13 and 16
Ancillary analyses	28	Any other analyses performed, including subgroup and sensitivity analyses, distinguishing pre-specified from post hoc	1 and 17
**Discussion**			
Interpretation	29	Interpretation consistent with results, balancing benefits and harms, and considering other relevant evidence	16-19
Limitations	30	Trial limitations, addressing sources of potential bias, imprecision, generalisability, and, if relevant, multiplicity of analyses	18-18

© 2025 Hopewell et al. This is an Open Access article distributed under the terms of the Creative Commons Attribution License (https://creativecommons.org/licenses/by/4.0/), which permits unrestricted use, distribution, and reproduction in any medium, provided the original work is properly cited.

*We strongly recommend reading this statement in conjunction with the CONSORT 2025 Explanation and Elaboration and/or the CONSORT 2025 Expanded Checklist for important clarifications on all the items. We also recommend reading relevant CONSORT extensions. See www.consort-spirit.org.

Table S2: Phytochemical characterization of microencapsulated persimmon (*Diospyros kaki* L.) and karonda (*Carissa carandas* L.) extracts

Phytochemical Components	Content (mg/g extract)
Total phenolic content (mg GAE)	15.7 ± 0.2
Anthocyanin content (mg cyanidin-3-O-glucoside)	0.4 ± 0.0
Gallic acid	2.1 ± 0.1
p-Coumaric acid	0.2 ± 0.0
Ferulic acid	0.7 ± 0.0
Rutin	0.5 ± 0.1
Quercetin	0.2 ± 0.0

Data are expressed as mean ± SD. Abbreviations: GAE, gallic acid equivalents

Table S3: Dietary energy and macronutrient intake of participants in each group before and after the intervention

	PLA (n = 11)		CBT (n = 10)		EATME (n = 11)		CBT+EATME (n = 11)		Time effect η^2 (p-Value)	Group×Time interaction η^2 (p-Value)
	Baseline	Post-test	Baseline	Post-test	Baseline	Post-test	Baseline	Post-test		
Energy intake (kcal/day)	2003.9 ± 168.3	1971.0 ± 166.2	1968.1 ± 160.1	2094.6 ± 193.7	1994.3 ± 228.8	2032.1 ± 234.0	2026.8 ± 211.1	2034.7 ± 237.8	0.030 (0.283)	0.077 (0.367)
Energy intake (kcal/kg/day)	33.5 ± 3.9	33.0 ± 4.3	32.7 ± 2.9	32.0 ± 3.0	33.7 ± 2.6	33.6 ± 2.2	35.7 ± 4.2	35.2 ± 4.3	0.068 (0.099)	0.020 (0.850)
Carbohydrate (g/day)	226.9 ± 28.9	211.9 ± 25.4	211.5 ± 32.3	213.8 ± 35.4	205.6 ± 36.8	214.1 ± 32.6	215.6 ± 26.1	211.9 ± 33.2	0.006 (0.629)	0.107 (0.213)
Carbohydrate (%TEI)	42.3 ± 5.0	43.2 ± 6.0	42.9 ± 5.0	41.0 ± 6.4	41.2 ± 5.7	42.3 ± 5.8	42.9 ± 6.4	42.3 ± 8.7	0.000 (0.897)	0.044 (0.620)
Protein (g/day)	135.8 ± 52.9	140.7 ± 47.9	130.5 ± 45.1	118.8 ± 34.7	116.3 ± 41.1	124.7 ± 47.9	124.4 ± 40.6	123.2 ± 40.3	0.000 (0.992)	0.020 (0.854)
Protein (%TEI)	25.0 ± 8.6	28.4 ± 8.9	26.7 ± 9.3	23.1 ± 7.8	23.3 ± 7.1	24.8 ± 9.7	24.5 ± 7.2	24.4 ± 7.9	0.001 (0.869)	0.057 (0.506)
Fat (g/day)	81.0 ± 6.0	81.2 ± 4.9	82.1 ± 7.4	81.3 ± 5.9	81.5 ± 5.9	79.5 ± 8.3	80.0 ± 7.4	80.6 ± 4.8	0.006 (0.618)	0.026 (0.787)
Fat (%TEI)	34.1 ± 3.2	37.3 ± 3.5	37.8 ± 4.5	35.3 ± 5.1	37.2 ± 5.0	35.8 ± 6.6	36.0 ± 5.8	36.1 ± 5.1	0.001 (0.888)	0.146 (0.101)

Data are expressed as mean ± SD. No significant differences in nutritional status were found at baseline between the groups, and no significant effects of time or the interaction between group and time were observed (all $p > 0.05$). Abbreviations: PLA, placebo control group; CBT, concurrent training group; EATME, microencapsulated polyphenol compounds supplementation group; CBT+EATME, combined concurrent training with microencapsulated polyphenol compounds supplementation group; TEI, total energy intake.

Table S4: Anthropometry and body composition parameters at baseline and after 8 weeks of intervention. #

	PLA (n = 11)		CBT (n = 10)		EATME (n = 11)		CBT+EATME (n = 11)		Time Effect η^2 (p-Value)	Group × Time interaction η^2 (p-Value)
	**Baseline**	**Post-test**	**Baseline**	**Post-test**	**Baseline**	**Post-test**	**Baseline**	**Post-test**		
BW (kg)	68.5 ± 14.2	68.6 ± 14.4	64.9 ± 10.3	62.0 ± 10.0^{a**}	67.0 ± 8.4	65.7 ± 9.2	63.4 ± 12.1	60.9 ± 14.5^{a**}	0.263 (0.001)††	0.150 (0.092)
BMI (kg/m^2)	26.5 ± 4.1	26.6 ± 4.3	25.9 ± 3.1	25.0 ± 4.0^{a*}	26.5 ± 3.8	25.9 ± 4.2	25.4 ± 5.0	24.4 ± 5.7^{a**}	0.246 (0.001)††	0.138 (0.118)
BF (%)	36.9 ± 7.1	37.2 ± 6.4	38.4 ± 6.4	37.5 ± 6.4	38.4 ± 6.6	39.6 ± 5.0	38.1 ± 7.3	36.7 ± 8.5	0.009 (0.550)	0.142 (0.110)
FM (kg)	24.1 ± 8.6	24.3 ± 8.4	23.7 ± 7.0	22.8 ± 6.5	24.8 ± 6.5	24.8 ± 6.4	23.1 ± 9.3	22.8 ± 9.3	0.056 (0.135)	0.121 (0.164)
FFM (kg)	42.9 ± 9.6	42.5 ± 9.1	39.5 ± 6.3	39.7 ± 6.7	41.6 ± 7.5	39.4 ± 4.5	37.8 ± 6.1	40.3 ± 9.0	0.000 (0.982)	0.121 (0.166)
LM (kg)	40.4 ± 9.3	40.1 ± 8.7	37.4 ± 6.0	37.6 ± 6.4	39.5 ± 7.2	37.2 ± 4.3	35.6 ± 5.8	36.2 ± 6.0	0.017 (0.410)	0.103 (0.233)
Arms LM (kg)	4.9 ± 1.7	4.8 ± 1.5	4.4 ± 0.9	4.4 ± 0.9	4.3 ± 1.0	4.0 ± 0.9	3.8 ± 0.8	4.2 ± 1.2	0.000 (0.907)	0.127 (0.147)
Legs LM (kg)	14.3 ± 3.9	14.0 ± 3.7	12.8 ± 2.5	13.1 ± 2.6	13.7 ± 3.0	12.9 ± 1.7	12.4 ± 2.4	13.4 ± 3.6	0.001 (0.810)	0.137 (0.120)
Android fat (%)	41.3 ± 11.0	43.4 ± 6.0	42.0 ± 8.3	41.0 ± 8.3	42.4 ± 8.8	44.1 ± 6.7	39.4 ± 11.1	38.8 ± 11.2	0.017 (0.411)	0.108 (0.213)
Gynoid fat (%)	38.2 ± 9.2	38.9 ± 8.8	41.9 ± 8.3	41.0 ± 8.4	41.0 ± 7.3	42.1 ± 5.3	41.7 ± 6.4	39.8 ± 7.8	0.007 (0.601)	0.135 (0.125)
A/G ratio	1.1 ± 0.3	1.2 ± 0.2	1.0 ± 0.2	1.0 ± 0.2	1.0 ± 0.2	1.1 ± 0.1	0.9 ± 0.2	1.0 ± 0.2	0.049 (0.163)	0.032 (0.735)

Data are expressed as mean ± SD. Abbreviations: PLA, placebo control group; CBT, concurrent training group; EATME, microencapsulated polyphenol compounds supplementation group; CBT+EATME, combined concurrent training with microencapsulated polyphenol compounds supple-mentation group; BW; body weight; BMI, body mass index; BF, body fat percentage; FM, fat mass; FFM, fat-free mass; LM, lean mass; A/G ratio, android to gynoid fat ratio. †† denotes a large effect size (partial $\eta^2 \geq 0.14$). * and ** indicate statistical significance at $p < 0.05$ and $p < 0.01$, respectively. Superscript a indicates a significant within-group difference from baseline.

Table S5: Renal and hepatic function markers #at baseline and after 8 weeks of intervention. #

	PLA (n = 11)		CBT (n = 10)		EATME (n = 11)		CBT+EATME (n = 11)		Time Effect η^2 (p-Value)	Group × Time interaction η^2 (p-Value)
	**Baseline**	**Post-test**	**Baseline**	**Post-test**	**Baseline**	**Post-test**	**Baseline**	**Post-test**		
BUN (mg/dL)	13.4 ± 3.8	13.2 ± 4.0	14.1 ± 4.6	12.8 ± 4.7	14.7 ± 4.2	12.3 ± 3.5	13.6 ± 4.7	13.8 ± 4.7	0.079 (0.075)	0.100 (0.246)
Creatinine (mg/dL)	0.7 ± 0.1	0.8 ± 0.2	0.8 ± 0.2	0.8 ± 0.1	0.7 ± 0.2	0.7 ± 0.2	0.9 ± 0.4	0.8 ± 0.4	0.002 (0.769)	0.040 (0.656)
eGFR (mL/min/1.73m^2)	103.3 ± 13.9	101.2 ± 15.3	90.6 ± 18.2	91.9 ± 15.7	101.1 ± 15.3	99.1 ± 12.9	99.8 ± 11.9	93.9 ± 23.6	0.033 (0.257)	0.046 (0.602)
AST (U/L)	29.1 ± 18.6	28.2 ± 12.0	19.2 ± 3.8	21.1 ± 4.2	23.1 ± 7.5	23.3 ± 8.4	24.3 ± 4.3	24.6 ± 5.9	0.003 (0.751)	0.014 (0.905)
ALT (U/L)	35.7 ± 38.9	39.1 ± 38.2	15.6 ± 4.0	17.5 ± 4.5	19.2 ± 11.9	17.4 ± 5.9	21.1 ± 4.0	22.3 ± 6.2	0.003 (0.730)	0.008 (0.954)
ALP (U/L)	69.0 ± 22.5	80.2 ± 32.5	76.9 ± 15.7	87.7 ± 20.5[a*]	70.9 ± 23.2	78.9 ± 22.1	73.6 ± 23.3	82.6 ± 23.6[a*]	0.321 (0.000)[††]	0.008 (0.111)

Data are expressed as mean ± SD. Abbreviations: PLA, placebo control group; CBT, concurrent training group; EATME, microencapsulated polyphenol compounds supplementation group; CBT+EATME, combined concurrent training with microencapsulated polyphenol compounds supplementation group; BUN, blood urea nitrogen; eGFR, estimated glomerular filtration rate; AST, aspartate aminotransferase; ALT, alanine aminotransferase; ALP, alkaline phosphatase. †† denotes a large effect size (partial $\eta^2 \geq 0.14$). * indicate statistical significance at $p < 0.05$, respectively. Superscript a indicates a significant within-group difference from baseline.

Table S6. Changes in quality of life parameters at baseline and after 8 weeks of intervention. #

	**PLA** (n = 11)		**CBT** (n = 10)		**EATME** (n = 11)		**CBT+EATME** (n = 11)		**Time Effect η^2 (p-Value)**	**Group × Time interaction η^2 (p-Value)**
	**Baseline**	**Post-test**	**Baseline**	**Post-test**	**Baseline**	**Post-test**	**Baseline**	**Post-test**		
Physical domain	24.6 ± 3.0	24.8 ± 3.0	23.9 ± 3.6	26.4 ± 3.8^{a**}	26.5 ± 2.8	27.2 ± 2.1	24.2 ± 3.1	26.6 ± 2.2^{a**}	0.264 (0.001)††	0.138 (0.119)
Psychological domain	22.3 ± 2.5	21.6 ± 2.3	22.3 ± 3.4	24.8 ± 2.9	23.5 ± 2.8	23.9 ± 3.7	22.0 ± 2.5	23.0 ± 2.1^{a**,b*}	0.105 (0.038)†	0.192 (0.038)††
Social domain	10.6 ± 1.9	10.6 ± 1.4	10.5 ± 1.8	11.5 ± 16	11.3 ± 1.8	11.1 ± 1.5	9.7 ± 1.9	10.9 ± 1.2	0.056 (0.137)	0.079 (0.357)
Environment	27.6 ± 2.6	27.1 ± 2.8	27.0 ± 4.0	29.9 ± 3.8^{a**}	30.1 ± 4.4	30.4 ± 3.5	26.2 ± 4.5	28.1 ± 4.6^{a*}	0.118 (0.028)†	0.158 (0.079)
Overall	91.6 ± 9.5	89.7 ± 9.6	90.1 ± 11.2	100.3 ± 10.1 ^{a**,b*}	94.6 ± 10.8	99.6 ± 9.4^{a*}	88.7 ± 8.5	95.2 ± 8.4^{a**}	0.366 (0.000)††	0.309 (0.002)††

Data are expressed as mean ± SD. Abbreviations: PLA, placebo control group; CBT, concurrent training group; EATME, microencapsulated polyphenol compounds supplementation group; CBT+EATME, combined concurrent training with microencapsulated polyphenol compounds supplementation group. † denotes a small-to-moderate effect size (0.06 ≤ partial η^2 < 0.14); †† denotes a large effect size (partial η^2 ≥ 0.14). * and ** indicate statistical significance at $p < 0.05$ and $p < 0.01$, respectively. Superscript a indicates a significant within-group difference from baseline; superscript b indicates a significant difference from the PLA group after 8 weeks of intervention.
